# Cloning and expression of a novel lactococcal aggregation factor from *Lactococcus lactis *subsp. *lactis *BGKP1

**DOI:** 10.1186/1471-2180-11-265

**Published:** 2011-12-19

**Authors:** Milan Kojic, Branko Jovcic, Ivana Strahinic, Jelena Begovic, Jelena Lozo, Katarina Veljovic, Ljubisa Topisirovic

**Affiliations:** 1 Laboratory for Molecular Genetics of Industrial Microorganisms, Institute of Molecular Genetics and Genetic Engineering, University of Belgrade, Vojvode Stepe 444/a, P.O. Box 23, Belgrade 11010, Serbia

## Abstract

**Background:**

Aggregation may play a main role in the adhesion of bacteria to the gastrointestinal epithelium and their colonization ability, as well as in probiotic effects through co-aggregation with intestinal pathogens and their subsequent removal. The aggregation phenomenon in lactococci is directly associated with the sex factor and lactose plasmid co-integration event or duplication of the cell wall spanning (CWS) domain of PrtP proteinase.

**Results:**

*Lactococcus lactis *subsp. *lactis *BGKP1 was isolated from artisanal semi-hard homemade cheese and selected due to its strong auto-aggregation phenotype. Subsequently, non-aggregating derivative (Agg^-^) of BGKP1, designated as BGKP1-20, was isolated, too. Comparative analysis of cell surface proteins of BGKP1 and derivative BGKP1-20 revealed a protein of approximately 200 kDa only in the parental strain BGKP1. The gene involved in aggregation (*aggL*) was mapped on plasmid pKP1 (16.2 kb), cloned and expressed in homologous and heterologous lactococci and enterococci. This novel lactococcal aggregation protein was shown to be sufficient for cell aggregation in all tested hosts. In addition to the *aggL *gene, six more ORFs involved in replication (*repB *and *repX*), restriction and modification (*hsdS*), transposition (*tnp*) and possible interaction with mucin (*mbpL*) were also located on plasmid pKP1.

**Conclusion:**

AggL is a new protein belonging to the collagen-binding superfamily of proteins and is sufficient for cell aggregation in lactococci.

## Background

The aggregation phenomenon in lactococci has been studied for more than thirty years. It was found to be directly associated with a sex factor and lactose plasmid co-integration event [1] or duplication of the cell wall spanning (CWS) domain of PrtP proteinase [2]. Lactose plasmid conjugation in *Lactococcus lactis *712 and in the related strains C2 and ML3, frequently involves plasmid co-integration with a sex factor. Moreover, this phenomenon is often associated with a cell aggregation phenotype and high frequency transfer ability [3-5]. The lactococcal sex factor exists integrated in the chromosome [6], although it can be excised as a closed circular form and lost from the cell [1]. Deletion and over-expression experiments confirmed that CluA is the only sex factor component responsible for aggregation in *L. lactis*. This 136 kDa surface-bound protein, encoded by the chromosomally located sex factor of *Lactococcus lactis *subsp. *cremoris *MG1363, is associated with cell aggregation linked to high-frequency transfer [7]. Two domains of CluA involved in distinct functions were determined. The region from D153-I483 is important for promoting cell-to-cell binding (aggregation), whereas K784-K1056 Tra domain is involved in DNA transfer and responsible for high conjugation frequency [8]. Furthermore, the aggregation ability of *L. lactis *subsp. *lactis *BGMN1-5 and its cured derivative was dependent on the presence of the plasmid encoded extracellular proteinase, PrtP [2,9]. The PrtP proteinase of BGMN1-5 contains a duplication of the C-terminal cell wall spanning domain (CWS). Experiments in which hybrids of BGMN1-5 PrtP, containing one or more CWS domains were constructed, showed that only cells producing a fusion protein with two or more CWS domains sedimented. Sedimentation resulted from specific interaction between CWS domains [2]. It is interesting that both, CluA protein and PrtP proteinase, have an LPXTG pentapeptide at the carboxy terminus, which is conserved among many cell surface proteins of Gram-positive bacteria [10]. In Gram-positive bacteria, these proteins have a multitude of functions, which include binding to host cells and/or tissues or specific immune system components, protein processing, nutrient acquisition and interaction between bacteria during conjugation [11]. Many cell-surface proteins are involved in aggregation and adhesion processes, including the colonization of oral and commensal bacteria [12-14] and initiation of infection by pathogens [15-19]. Pathogenic Gram-positive bacteria express cell surface proteins that contribute to virulence [20]. The genes encoding the surface proteins derived from several *Enterococcus faecalis *plasmids, including pAD1, pPD1 and pCF10 have been sequenced [21-23] and over-expressed in different bacteria including *Lactococcus lactis *[24]. It was found that aggregation substance (AS), a surface protein of *E. faecalis*, might contribute to virulence [25].

*L. lactis *subsp. *lactis *BGKP1 was selected in our laboratory as a specific strain due to the expression of a unique auto-aggregation phenotype. In the present study, we isolated a non-aggregating derivative (Agg^-^) of BGKP1 and performed comparative analysis. We found that a cell surface protein of high molecular mass, around 200 kDa, is responsible for the aggregation. The gene encoding for aggregation protein (*aggL*) was mapped on plasmid pKP1 (16.2 kb). The gene was cloned, sequenced and expressed in homologous and heterologous lactococcal and enterococcal hosts, showing that AggL protein is responsible for cell aggregation in lactococci. Therefore, we propose AggL as a novel lactococcal aggregation factor.

## Results and Discussion

Aggregation may play the main role in adhesion of bacteria to the gastrointestinal epithelium and their colonization ability, as well as in probiotic effects through co-aggregation with intestinal pathogens and their subsequent removal.

### Isolation and comparative analyses of *Lactococcus lactis *subsp. *lactis *BGKP1 and its non-aggregating derivative BGKP1-20

Considering the importance of aggregation, *Lactococcus lactis *subsp. *lactis *BGKP1 was selected during the characterization of microflora of artisanal white semi-hard homemade cheeses manufactured in the village of Rendara (altitude 700 m) on Kopaonik mountain, Serbia. Among 50 lactic acid bacteria (LAB), *Lactococcus lactis *subsp. *lactis *BGKP1 was chosen for further study due to its strong auto-aggregation phenotype (Agg^+^).

**Figure 1:**
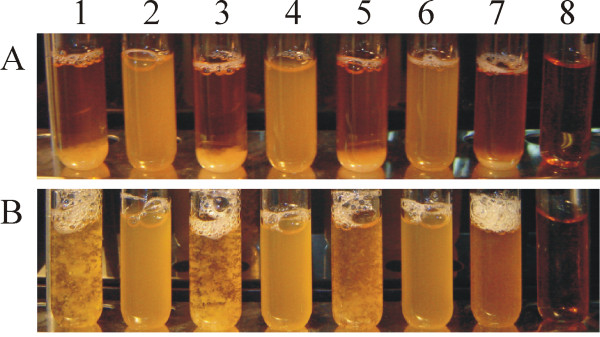
**Aggregation ability of *L. lactis *subsp. *lactis *BGKP1, BGKP1-20 and transformants carrying pAZIL-KPPvSc1 in growth medium after overnight cultivation (A) and vigorous mixing (B)**. 1. *L. lactis *subsp. *lactis *BGKP1 (Agg^+^); 2. *L. lactis *subsp. *lactis *BGKP1-20 (Agg^-^); 3. *L. lactis *subsp. *lactis *BGKP1-20/pAZIL-KPPvSc1; 4. *L. lactis *subsp. *cremoris *MG1363; 5. *L. lactis *subsp. *cremoris *MG1363/pAZIL-KPPvSc1; 6. *L. lactis *subsp. *lactis *BGMN1-596; 7. *L. lactis *subsp. *lactis *BGMN1-596/pAZIL-KPPvSc1; 8. GM17 medium.

BGKP1 is a lactose positive, bacteriocin and proteinase non-producing strain. The aggregation phenotype may be observed after vigorous mixing of a stationary phase culture, when snowflake-like aggregates become visible (Figure 1). The aggregates of BGKP1 cells differed in appearance from those of *L. lactis *subsp. *cremoris *MG1363 expressing CluA or *L. lactis *subsp. *lactis *BGMN1-5. Aggregates rapidly sedimented under resting conditions and more than 95% of BGKP1 cells aggregated in the first minute, as observed by the decrease of cell suspension absorbance (data not shown). BGKP1 cell aggregates resemble those of *Lactobacillus paracasei *subsp. *paracasei *BGSJ2-8 [26]. The aggregation ability of BGKP1 was lost spontaneously after transfer of cells from -80°C to 30°C, with a frequency of 5% to 10%, as previously shown for BGSJ2-8 [26]. The resulting non-aggregating derivative (Agg^-^) of BGKP1 was designated as BGKP1-20. Agg^+ ^cells formed smaller and prominent colonies, whereas Agg^- ^derivatives showed flat colonies on agar plates. Mutations in genes encoding biofilm-associated proteins were also shown to result in transformation of colony morphology [27]. Since BGKP1 and BGKP1-20 were not able to form biofilms on plastic tissue culture plates, the aggregation phenomenon present in BGKP1 is most probably not linked to biofilm formation. Spontaneous high-frequency loss of the trait indicated a plasmid location of the gene(s) encoding the aggregation phenotype. Comparative analysis of the fitness of BGKP1 and BGKP1-20 in the exponential phase of growth showed differences in generation times between the strains. The doubling time for BGKP1 was 54.4 min (specific growth rate = 1.103/h), while that for BGKP1-20 was 50.2 min (specific growth rate = 1.195/h). The presence of the aggregation phenotype resulted in a significantly prolonged doubling time for BGKP1 (approximately 8.5%) when compared with that of BGKP1-20. Taking into consideration that bacteria maintain and procure gene coding for the aggregation factor in spite of the energy cost, we could hypothesize that this feature provides some benefit for the cell.

### Nature of molecules involved in aggregation

The spontaneous loss of the capacity to aggregate in BGKP1 was tested under various conditions. Aggregation capacity was found to be reversibly lost after repeated washing of BGKP1 cells with bi-distilled water. Nevertheless, when washed BGKP1 cells that had lost the Agg^+ ^phenotype were re-suspended in the wash material, they re-gained the ability to aggregate. Obviously, a some molecule(s) with a role in aggregation were washed from the cell wall. However, aggregation was not observed when BGKP1-20 Agg^- ^cells were re-suspended in wash material from BGKP1 Agg^+^. To check the nature of molecules involved in the aggregation, BGKP1 Agg^+ ^cells were treated with proteinase K prior to washing by water. The wash material of proteinase K-treated cells did not restore the aggregation ability of BGKP1 Agg^- ^washed cells. Results indicated that the aggregation factor is of proteinaceous nature. Since a protein is involved in aggregation, the influence of various pH levels and the concentration of five ions (K^+^, Na^+^, Ca^++^, Mg^++ ^and Fe^+++^) on this phenomenon was examined. It was found that pH did not have as strong impact on the ability of BGKP1 to aggregate as cations did, especially iron. The presence of 1 mM FeCl_3 _promoted aggregation of BGKP1 washed cells.

**Figure 2:**
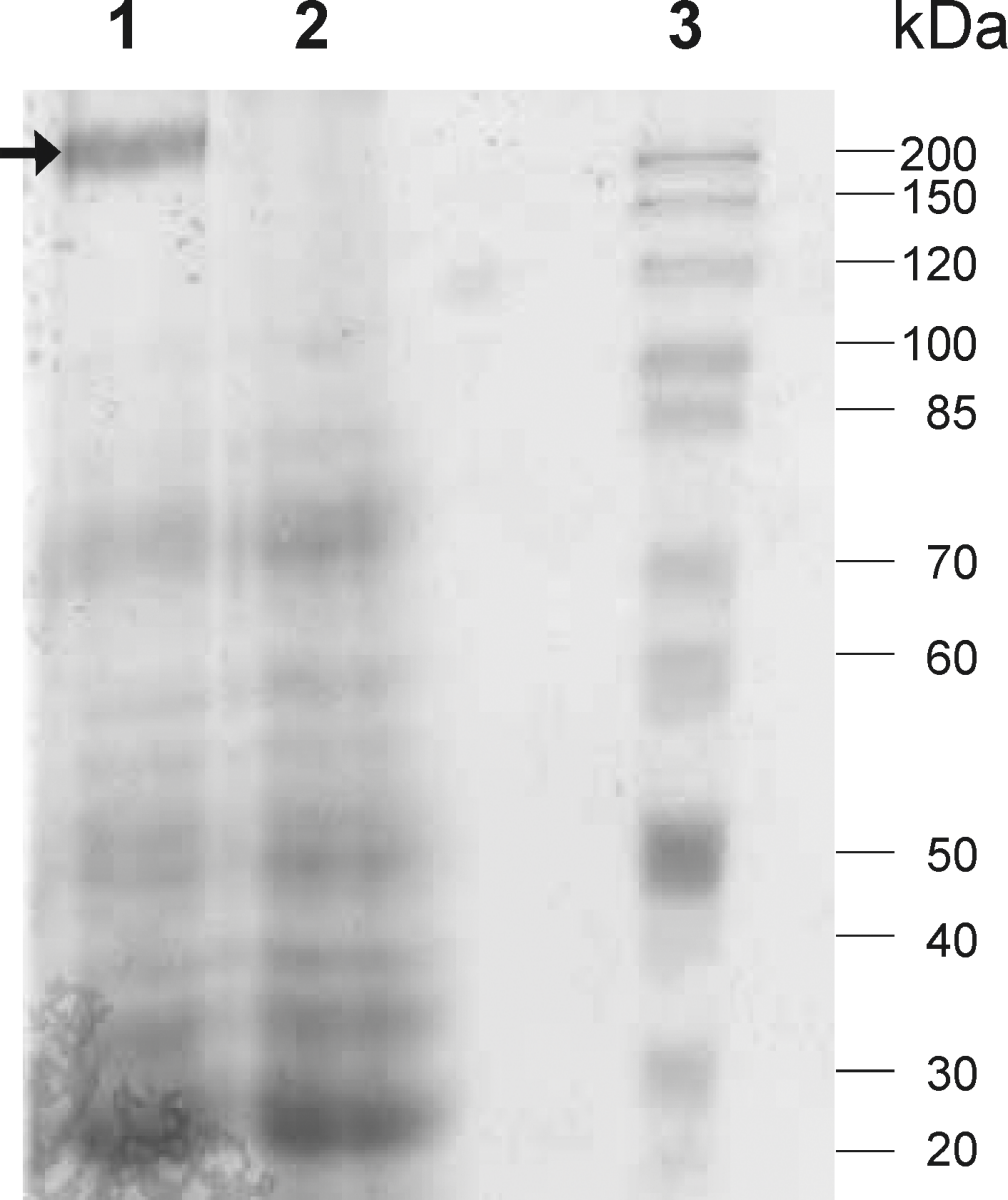
**SDS-PAGE of cell-surface proteins isolated from *L. lactis *subsp. *lactis *BGKP1 and BGKP1-20**. Lane 1. BGKP1 Agg^+^; Lane 2. BGKP1-20 Agg^- ^derivative; Lane 3. Molecular marker - protein ladder from 10 to 200 kDa (Fermentas, Vilnius, Lithuania). Arrow indicates high molecular-mass protein band present only in Agg^+ ^strain.

Cell surface protein profiles of BGKP1 and the Agg^- ^derivative BGKP1-20 were compared in order to detect any differences between strains. As demonstrated for BGSJ2-8 [26], the SDS-PAGE pattern of cell surface proteins from BGKP1 and BGKP1-20 differed. Thus, Agg^+ ^contained an additional ≈200 kDa protein, which was absent from the BGKP1-20 Agg^- ^derivative (Figure 2). This suggested that the aforementioned protein might be responsible for the aggregation. The protein detected and potentially involved in the aggregation of *L. lactis *subsp. *lactis *BGKP1 had a slightly smaller molecular mass than that of *L*. *paracasei *subsp. *paracasei *BGSJ2-8.

### Localization and cloning of genes linked to the aggregation phenomenon

**Figure 3:**
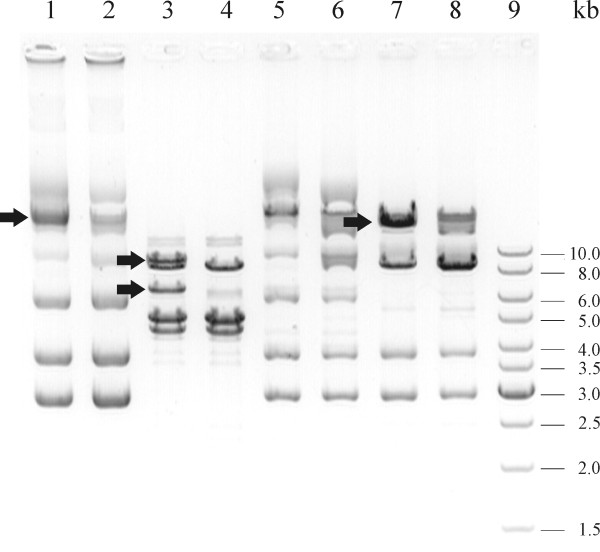
**Plasmid profiles of *L. lactis *subsp. *lactis *BGKP1 Agg^+ ^(Lanes 1, 3, 5 and 7) and BGKP1-20 Agg^- ^derivative (Lanes 2, 4, 6 and 8) analysed on 1% agarose gel**. Lanes 1 and 2, non-digested plasmids; Lanes 3 and 4, plasmids digested with *Eco*RI restriction enzyme; Lanes 5 and 6, plasmids digested with *Pst*I restriction enzyme; Lanes 7 and 8, plasmids digested with *Sal*I restriction enzyme; Lane 9. Gene Ruler DNA size marker (Fermentas, Vilnius, Lithuania). Arrows indicate positions of plasmid bands/fragments present only in *L. lactis *subsp. *lactis *BGKP1 Agg^+ ^strain.

Plasmid profile analysis (of non-digested and digested plasmids with different restriction enzymes) of parental strain BGKP1 and the Agg^- ^derivative BGKP1-20 showed differences in one plasmid designated as pKP1, indicating its potential role in the expression of the aggregation phenotype (Figure 3).

In order to facilitate cloning and expression of gene(s) responsible for the aggregation phenotype in homologous and heterologous hosts, new lactococcal-*E. coli *shuttle cloning vectors pAZIL and pAZILcos, based on pACYC184 [28] and pIL253 [29] were constructed [see Additional File 1]. These vectors enabled cloning of large DNA fragments (entire pKP1 - 16.2 kb), blue-white selection for the inserted fragments and high stability of the constructs. The plasmid library of pKP1 constructed in pAZIL enabled sequencing and subsequent *in silico *analysis of the obtained sequence.

### Sequence analyses of plasmid pKP1

**Figure 4:**
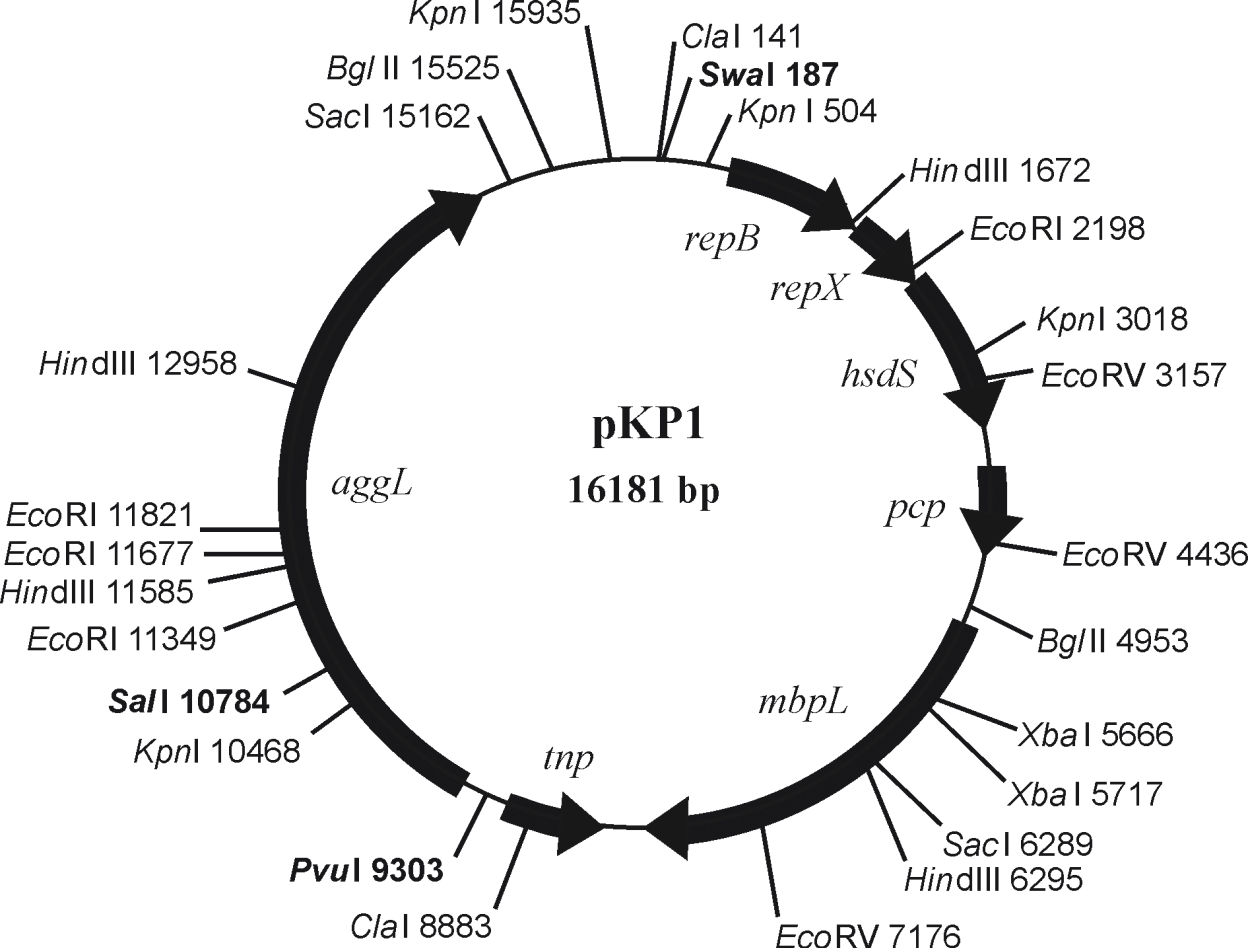
**Circular map of *L. lactis *subsp. *lactis *BGKP1 plasmid pKP1 with ORFs and positions of restriction enzyme sites**. Restriction enzymes with a single recognition site are given in bold.

The complete sequence of plasmid pKP1 was found to consist of 16181 bp, with a G+C content of 35.94%. Within the 4380 bp long nucleotide sequence of pKP1 (region 15394-1-3593), a 99% identity with the pSRQ900 plasmid of *Lactococcus lactis *(GenBank Accession No. AF001314) was determined. This sequence represented approximately one fourth of the pKP1 nucleotide sequence. This region encompassed the origin of replication, *repB *gene, *repX *replication associated gene and putative *hsdS *gene (Figure 4). The rest of the nucleotide sequence (three quarters of pKP1) did not share identity with pSRQ900 and carried three genes, including two new genes (*aggL *and *mbpL*) and one known transposase gene, which implies its novelty.

In addition, seven open reading frames (ORF) were revealed in pKP1 by application of the DNA Strider program (Table 1, Figure 4). It is interesting that all ORFs on the plasmid were in the same orientation except the gene encoding for transposase. Two potential ORFs, designated as *aggL *and *mbpL*, were additionally analysed. BLAST search revealed that *aggL *gene showed no similarity with any of the genes from the NCBI BLAST database, while AggL protein shared 51% identity with a hypothetical protein from *Oenococcus oeni *AWRIB429 (Table 1). The nucleotide sequence of *mbpL *shared 84% identity with ORF from *Leuconostoc citreum *KM20 plasmid pLCK1 and 53% amino acid identity with a protein from *Enterococcus faecalis *TX1322 (Table 1). Motif Scan http://myhits.isb-sib.ch/cgi-bin/motif_scan and DAS Transmembrane Domain [30] programs were used to analyse their potential protein products. It was revealed that AggL included several motifs important for cell adhesion, such as a collagen-binding domain with a jelly-roll fold (C-terminus), CnaB-like domain (C-terminus) as well as serine and threonine-rich domains (N-terminus). MbpL contained a MucBP-like domain and YSIRK-signal. Both AggL and MbpL were predicted to have the Gram-positive cocci cell wall anchoring domain (LPXTG) and two transmembrane domains (by using strict cutoff). Additionally, both proteins had short amino acid repeat regions at the N-terminus, serine and threonine rich regions for AggL and an alanine rich region for MbpL. MbpL had five identical consecutive repeats at its N-terminus, each encompassing 26 aa (AETASSSSSS AVKAETTSAS SSSAVK) starting at position 71 and ending at position 200, and two identical repeats at its C-terminus consisting of 36 aa (GDSYTTEQKA IPGYTFKAVQ GNPTGQFTSD AQTVTY), the first at position 750-785 and the second at 890-925. At its C-terminus, AggL protein encompassed four repeats of 70 aa (NTHQVAKTSV SGQKTWSDHD NQDGLRPDEI TVNLLADGKK VDSKTVTAKD GWKYEFNDLD KFKAGQEIKY) organised in two pairs with a space of 21 aa between repeats and 118 aa between pairs (repeat positions: I-1241-1310; II-1331-1400; III-1518-1587 and IV-1608-1677) [see Additional file 2].

**Table 1:** General features of putative ORFs from pKP1 with best matches to sequences in the public database

Protein or gene	Position	Size (nc/aa)	Proposed function	Source strain	% of identity (nc/aa)	GenBank accession no. (nc/aa)
RepB	600-1760	1161/386	replication protein	*Lactococcus lactis *plasmid pSRQ900	99/99	AF001314.1/NP_862549.1
RepX	1757-2344	588/195	replication associated protein	*Lactococcus lactis *plasmid pSRQ900	100/100	AF001314.2/NP_862550.1
HsdS	2320-3510	1191/396	LldI type R/M, specificity subunit (HsdS)	*Lactococcus lactis *plasmid pSRQ900	100/100	AF001314.2/NP_862551.1
*pcp*	3821-4468	648/215	pyrrolidone-carboxylate peptidase	*Lactococcus lactis *plasmid pSK11P	99/99	DQ149245.1/ABA43397.1
*mbpL*	5022-8018	2997/998	mucin-binding domain protein	*Leuconostoc citreum *KM20 plasmid pLCK1/*Enterococcus faecalis *TX1322	84/53	DQ489740.1/ZP_04433966.1
Tnp	9170-8484	687/228	IS1216 transposase	*Enterococcus faecalis *strain EF-01 plasmid pEF-01/*Enterococcus faecalis*	99/99	CP002208.2/YP003896017.1
*aggL*	9526-14829	5304/1767	lactococcal aggregation factor	No similarity/*Oenococcus oeni *AWRIB429.	-/51	-/ZP06554154.1

nc - nucleotide

aa - amino acid

Primary structural analysis of AggL revealed domain organization similar to LPXTG proteins of Gram-positive cocci. The LPXTG motif is a highly conserved part of the C-terminal sorting signal and it plays a role in the covalent linkage of many cell-wall-associated surface proteins to the nascent pentaglycine crossbridge in peptidoglycan [22]. For example, *S. aureus *is known to express 21 proteins with the LPXTG motif including two clumping factors ClfA and ClfB [20,31]. Another characteristic of AggL primary protein structure is modular architecture and a number of repeat regions that share high mutual identity (98-100%). Previous studies on staphylococcal LPXTG proteins indicated modular architecture and B repeats as their specific characteristics. Such organization could have arisen during evolution through the acquisition of distinct domain-sized polypeptides of which some have expanded by duplication and homologous recombination [31]. Collagen-binding protein B domain (CnaB domain) is the most abundant domain of AggL. Such a structure might mediate bacterial adherence to collagen. Repeated units have been suggested to serve as a 'stalk' that projects the region crucial for adherence to the bacterial surface, thus facilitating bacterial adherence to collagen. Additionally, the N-terminal serine and threonine rich domains of AggL could play a role in aggregation, since it is known that such domains of CD46 protein promote efficient adherence of *Neisseria gonorrhoeae *to host cells [32]. Interestingly, the YSIRK domain, another characteristic of staphylococcal LPXTG proteins, was not found in AggL, although it was present within the signal peptide of MbpL. The requirement of a YSIRK motif for efficient secretion implies the existence of a specialized mode of substrate recognition by the secretion pathway of Gram-positive cocci. However, this mechanism is not essential for the surface protein to anchor to the cell wall envelope [33]. Considering the primary protein organization of MbpL, its role in the cell could most likely be interaction with gastrointestinal epithelial cells. Interestingly, the search for lactococcal proteins similar to AggL and MbpL against the NCBI BLAST database revealed that AggL shared identity only within its N-terminal region (encompassing transmembrane domain, serine and threonine rich domains, collagen binding domain and WD repeats). On the other hand, MbpL shared identity within its C-terminal region (encompassing the MucBP-like domain including 36 aa repeats, the transmembrane domain and the G+ anchoring domain).

### Homologous and heterologous expression of the *aggL *gene

**Figure 5:**
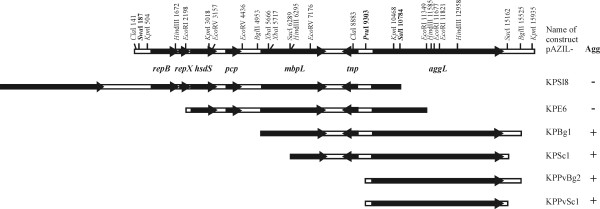
**Linear physical map of pKP1 and the scheme of constructed clones in the pAZIL cloning vector used for homologous and heterologous expression of aggregation phenotype**. Relevant restriction sites are indicated. Restriction enzymes with a single recognition site are given in bold. Bold arrows indicate the size and orientation of predicted ORFs. + - construct with aggregation ability; - - construct with no aggregation ability.

Cloning of different fragments of plasmid pKP1 into the pAZIL vector (Figure 5) and transfer of constructs into three lactococcal strains (homologous Agg^- ^derivative BGKP1-20, and heterologous MG1363, BGMN1-596 and *Enterococcus faecalis *BGZLS10-27) revealed that the *aggL *gene is sufficient for the aggregation phenotype (Figure 1). Furthermore, aggregation of enterococcal cells carrying the *aggL *gene was observed, but the intensity of cell aggregation was lower than that obtained in lactococci (data not shown).

This conclusion was confirmed by transformation of the same lactococci with two types of constructs: pAZIL harboring pKP1 linearized in the *aggL *gene, that results in the inactivation of this gene (construct pAZIL-KPSl8) and by constructs carrying the DNA fragment of pKP1 containing solely the *aggL *gene (for example pAZIL-KPPvSc1) (Figure 5). It was noticed that cell aggregation phenotypes of MG1363 and BGKP1-20 transformants, carrying the *aggL *gene, were identical to those of the parental strain BGKP1. Transformants of BGMN1-596 showed the aggregation phenotype with slightly different cell aggregates, which were smaller than in BGKP1 (Figure 1).

The location of the gene involved in the aggregation of BGKP1 on plasmid pKP1 potentially enables transfer of this factor through the microbial population.

Experiments with heterologous expression of *aggL *and/or *mbpL *revealed the main role of AggL protein in the aggregation phenomena. According to the morphological characteristics of cell aggregates in heterologous strains, we can assume that even though AggL is crucial for aggregation, some additional protein(s) (like MbpL) might have a modulatory effect on the aggregation phenotype. Additionally, preliminary *ex vivo *experiments with rat colon sections indicated that AggL is not involved in adhesion to the gastrointestinal epithelium (data not shown).

Further experiments will be focused on studies of AggL and MbpL interactions with human epithelial cells and their role in the adhesion and possible probiotic potential of BGKP1. Moreover, co-aggregation with various pathogenic bacteria will be also tested.

## Conclusions

We have demonstrated that in lactococci, a novel aggregation-promoting factor AggL is encoded by the *aggL *gene located on the 16.2 kb pKP1 plasmid. Moreover, functionality of *aggL *was confirmed by homologous and heterologous expression of different clones containing or lacking this gene in the newly constructed shuttle-cloning vector, pAZIL.

## Methods

### Bacterial strains, media, growth conditions and transformations

*Lactococcus lactis *subsp. *lactis *BGKP1 (Agg^+^) was isolated from semi-hard homemade cheese using standard microbiological procedures. Preliminary strain classification was done according to its fermentation ability using API 50CHL (Api System SA; Bio-Merieux, Montelieu-Vercieu, France), temperature of growth (30°C, 37°C, and 45°C), growth in the presence of salt (4% and 6.5%) and pH tolerance. Further taxonomic classification of BGKP1 involved repPCR with (GTG)_5 _primer [34], and sequencing of amplified 16S rDNA [35]. A non-aggregating derivative BGKP1-20 (Agg^-^), *L. lactis *subsp. *lactis *BGMN1-596 (9), *L. lactis *subsp. *cremoris *MG1363 [36] and *Enterococcus faecalis *BGZLS10-27 [37] were used for homologous and heterologous expression of the aggregation phenotype. Lactococcal and enterococcal strains were grown at 30°C in M17 medium [38] supplemented with 0.5% glucose (GM17) and stored in the same medium containing 15% (w/v) glycerol (Sigma Chemie GmbH, Deisenhofen, Germany) at -80°C. *L. lactis *and *E. faecalis *electrocompetent cells were prepared and transformed as previously described [39] using an Eppendorf Electroporator (Eppendorf, Hamburg, Germany). *E. coli *strain DH5α [40] was used for cloning experiments and plasmid propagation. DH5α was grown at 37°C in Luria-Bertani (LB) medium [41]. Agar plates were prepared by addition of agar (1.5% w/v) to the corresponding broth. *E*. *coli *competent cells were prepared using chemical treatment and subjected to heat shock transformation. Transformants were selected using the antibiotic erythromycin (5 μg ml^-1 ^for lactococci and enterococci or 250 μg ml^-1 ^for *E. coli*). Bacteriocin and proteinase activity were determined as described previously [9].

### Growth kinetics

Cultures of BGKP1 and BGKP1-20 were grown in 10 ml of GM17 to a density of 10^9 ^cells ml^-1^. Approximately 10^6 ^cells of each strain were added to 10 ml of GM17 and cultures were incubated at 30°C. Aliquots from each culture were taken every hour and plated on solid GM17 medium to calculate generation time for each strain. Experiments were done in triplicate.

### Molecular techniques

Molecular cloning techniques like end filling of DNA fragments with the Klenow fragment of DNA polymerase, dephosphorylation, ligation, PCR amplification and agarose gel DNA electrophoresis were carried out essentially as described previously [41]. The mini-prep method [42] was used for isolation of large plasmids from lactococci. Plasmids from *E. coli *were isolated using the QIAprep Spin Miniprep kit according to the manufacturer's recommendations (QIAGEN, Hilden, Germany). The DNA fragments from agarose gels were purified using a QIAqick Gel extraction kit as described by the manufacturer (QIAGEN). Digestion with restriction enzymes was conducted according to the supplier's instructions (Fermentas, Vilnius, Lithuania).

### Determination of the effect of ions, pH and proteinase K on aggregation ability of *L. lactis *subsp. *lactis *BGKP1

The effect of different ions and pH values on the BGKP1 aggregation phenotype was tested using cells that were three times washed in bi-distilled water until the aggregation phenotype was lost. Cells were then resuspended in buffers of different pH and solutions of various ions. The following buffers were used: KCl (pH 3.0), HCl-glycine (pH 3.0), Na-citrate (pH 4.0 to 6.0), Tris-HCl (pH 7.0 to 10.0) and Tris-NaOH (pH 11.0 to 12.0). The following ions were examined: K^+^, Na^+^, Ca^++^, Mg^++ ^and Fe^+++ ^in concentrations of 0.1, 1, and 10 mM. Proteinase K (1 μg ml^-1^) treatment was done in TE (10 mM Tris, 1mM EDTA, pH8) buffer for 1 h at 37°C. Determination of aggregation phenotype was based on absorption at 600 nm.

### Biofilm formation

The ability of BGKP1 and BGKP1-20 to form biofilms was tested as previously described by Christensen and coauthors [43]. *Pseudomonas aeruginosa *PAO1 and *Escherichia coli *DH5α were used as the positive and negative control strains respectively. The experiments were done in triplicate.

### Analysis of cell surface proteins of *L. lactis *subsp. *lactis *BGKP1 and its non-aggregating derivative

Cells from overnight culture (250 ml) of strain BGKP1 and its Agg^- ^derivative BGKP1-20 were harvested by centrifugation and washed in 50 ml bi-distilled water. Proteins from the wash were precipitated with ammonium sulphate (25% saturation). Precipitated proteins were resuspended in 10 mM Tris-HCl, pH 8.5, and applied on SDS-PAGE (10%). The obtained bands were visualized by Coomassie blue staining.

### Construction of shuttle-cloning vectors

The pAZIL shuttle-cloning vector and pAZILcos cosmid vector were constructed in order to perform the molecular analysis of BGKP1 plasmid pKP1 [see Additional File 1]. The tetracycline resistance gene of pACYC184 was replaced with the *lacZ *gene from the replicative form of M13 mp18 phage using *Cla*I/*Nar*I and *Hinc*II/*Ava*II restriction enzymes, resulting in cloning vector pAZ1. In the next step, the chloramphenicol resistance gene from pAZ1 was removed using *Sca*I and *Xmn*I restriction enzymes and the vector was fused with lactococcal cloning vector pIL253, previously digested with *Eco*RI-*Xba*I restriction enzymes and blunted with Klenow enzyme, resulting in shuttle cloning vector pAZIL.

To obtain a cosmid vector for the construction of cosmid libraries of lactococcal genomes, the *cos *site was introduced into the unique *Sac*II (7697) restriction site of the pAZIL vector. The DNA fragment containing the *cos *site was obtained by PCR amplification with primers cosF-CATGTTTGACCGCGGATCATCG and cosR-CTAGACACCGCGGAAGCTAGC (*Sac*II restriction sites are underlined). Afterwards, the PCR amplicon was digested with *Sac*II and ligated with *Sac*II-digested pAZIL resulting in the pAZILcos cosmid vector.

### Construction of various plasmid pKP1 derivatives

Strain BGKP1 harbors at least three plasmids. Total plasmids isolated from strain BGKP1 were digested with different restriction enzymes (*Sal*I, *Eco*RI, *Bgl*II, *Sac*I, *Pvu*I and *Bgl*II, *Sac*I and *Pvu*I). The resulting fragments were cloned into pAZIL vector digested with the same restriction enzymes (except for *Bgl*II, which was cloned into *Bam*HI) and selected in *E. coli *DH5α by the blue/white color method on LB plates containing erythromycin (250 μg ml^-1^), IPTG (0.1 mM) and X-Gal (40 μg ml^-1^). The obtained constructs carrying fragments of the largest plasmid pKP1 were designed as pAZIL-KPSl8 (16181 bp pKP1 plasmid linearized in *Sal*I at position 10784 resulting in a disrupted *aggL *gene), pAZIL-KPE6 (9151 bp *Eco*RI fragment of pKP1, position 2198-11349), pAZIL-KPBg1 (10572 bp *Bgl*II fragment of pKP1, position 4953-15525), pAZIL-KPSc1 (8873 bp *Sac*I fragment of pKP1, 6289-15162), pAZIL-KPPvBg2 (6322 bp *Pvu*I-*Bgl*II fragment of pKP1, position 9303-15525), and pAZIL-KPPvSc1 (5859 bp *Pvu*I-*Sac*I fragment of pKP1, 9303-15162). Restriction enzyme digestion and sequencing of the constructs were performed to show that the anticipated final plasmid constructs had been obtained. The constructs were isolated from *E. coli *and then transferred to *L. lactis *subsp. *lactis *BGKP1-20 (Agg^-^), *L. lactis *subsp. *lactis *BGMN1-596 and *L. lactis *subsp. *cremoris *MG1363 by electroporation. The obtained Em^r ^transformants were tested for expression of the aggregation phenotype.

### DNA sequencing and analysis

For DNA sequencing, pAZIL-KPSl8 and the other constructs aforementioned were isolated from *E. coli *using a QIAprep Spin Miniprep Kit (QIAGEN) as recommended by the manufacturer. Plasmids were sequenced by primer-walking of both strands in Macrogen's sequencing service (Seoul, Korea). Sequence annotation and the database search for similar sequences were performed using BLAST site programs at the National Center for Biotechnology Information [44]. The DNA Strider program was used for open reading frame (ORF) prediction.

### Nucleotide sequence accession number

The nucleotide sequences of the partial 16S rDNA sequence of *L. lactis *subsp. *lactis *BGKP1, plasmids pAZILcos and pKP1 were submitted to the EMBL GenBank under accession numbers FR873574, FR872379 and FR872378, respectively.

## Authors' contributions

MK was responsible for the conception and design of the study, and was involved in construction of shuttle-cloning vectors, pKP1 plasmid cloning and sequencing as well as in writing the draft and final version of the manuscript. BJ performed the experiments to analyse cell surface proteins and the effects of ions, pH and proteinase K on aggregation ability of the analysed strains, and was involved in sequencing and *in silico *analysis of pKP1 plasmid. IS participated in construction of plasmid pKP1 derivatives. JB was involved in construction of pAZ1, pAZIL and pAZILcos vectors and interpretation of data. JL participated in homologous and heterologous expression of aggregation phenotype. KV carried out plasmid profile analysis and standardization of transformation protocols. LT critically revised the manuscript and gave final approval of the version to be published. All authors read and approved the final manuscript.

## Supplementary Material

Additional file 1 **Construction strategy and the restriction enzyme maps of the lactococci/*E. coli *shuttle-cloning and cosmid vectors, pAZIL and pAZILcos**. pAZIL shuttle-cloning vector was constructed in the following order: tetracycline resistance gene of pACYC184 was replaced with the *lacZ *gene from the replicative form of M13 mp18 phage using *Cla*I/*Nar*I and *Hinc*II/*Ava*II restriction enzymes, resulting in cloning vector pAZ1. Subsequently chloramphenicol resistance gene from pAZ1 was removed using *Sca*I and *Xmn*I restriction enzymes and the vector was fused with lactococcal cloning vector pIL253, resulting in shuttle cloning vector pAZIL. Cosmid vector pAZILcos was obtained by introduction of *cos *site into the unique *Sac*II (7697) restriction site of the pAZIL vector. Only relevant restriction enzymes are shown. Restriction enzymes with a single recognition site are given in bold. Click here for file

Additional file 2 **Schematic presentation of AggL and MbpL proteins**. Boxes indicate domains of proteins and arrows indicate repeats. Click here for file
