# Supplementary figures and images for: Cloning and expression of a novel lactococcal aggregation factor from Lactococcus lactis subsp. lactis BGKP1
Source: BMC Microbiol. 2011 Dec 19;11:265. doi: 10.1186/1471-2180-11-265 (PMC3282668; doi:10.1186/1471-2180-11-265)

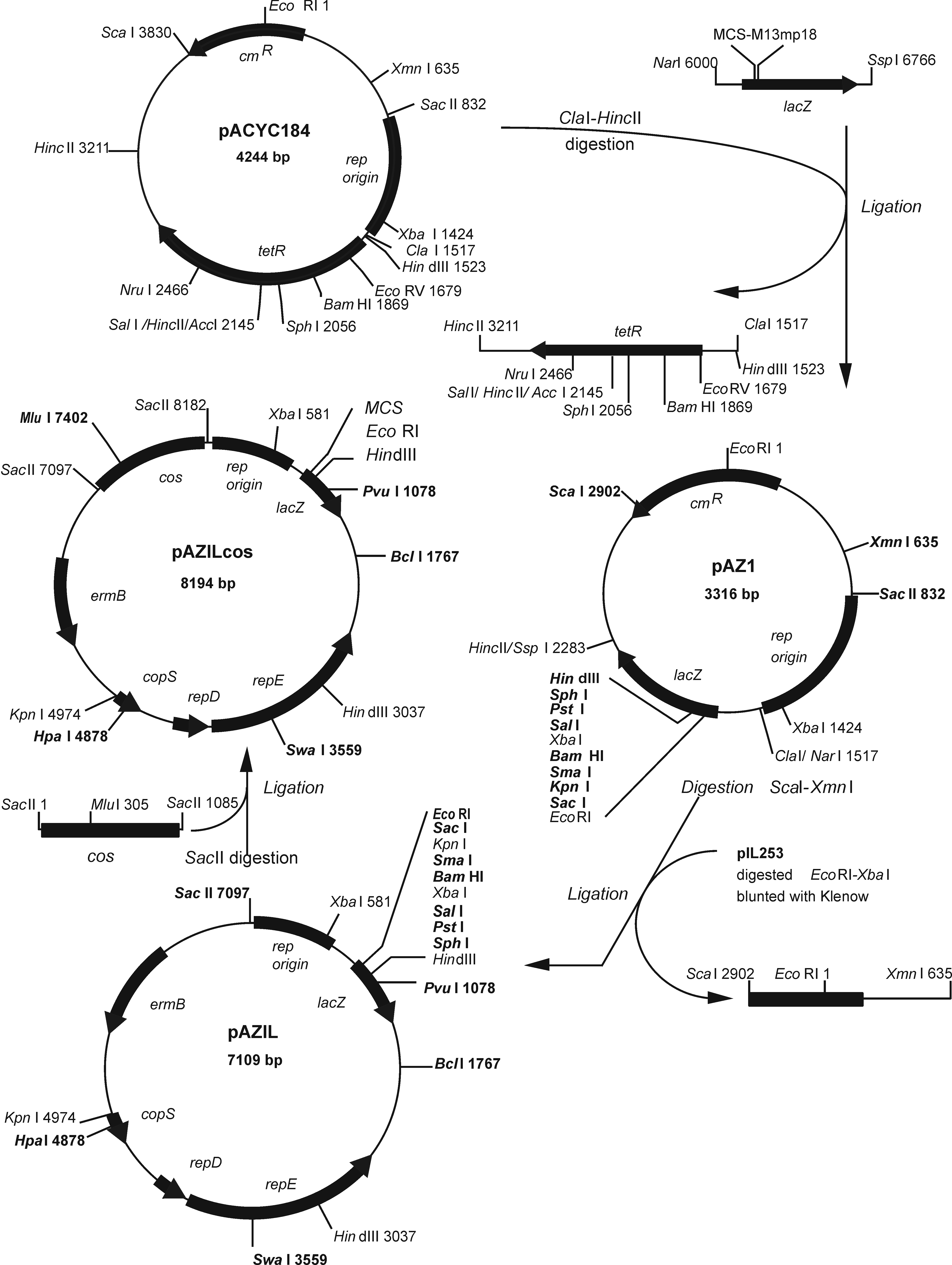

Supplement: Additional file 1 — Construction strategy and the restriction enzyme maps of the lactococci/E. coli shuttle-cloning and cosmid vectors, pAZIL and pAZILcos. pAZIL shuttle-cloning vector was constructed in the following order: tetracycline resistance gene of pACYC184 was replaced with the lacZ gene from the replicative form of M13 mp18 phage using ClaI/NarI and HincII/AvaII restriction enzymes, resulting in cloning vector pAZ1. Subsequently chloramphenicol resistance gene from pAZ1 was removed using ScaI and XmnI restriction enzymes and the vector was fused with lactococcal cloning vector pIL253, resulting in shuttle cloning vector pAZIL. Cosmid vector pAZILcos was obtained by introduction of cos site into the unique SacII (7697) restriction site of the pAZIL vector. Only relevant restriction enzymes are shown. Restriction enzymes with a single recognition site are given in bold. [file 1471-2180-11-265-S1.TIFF]

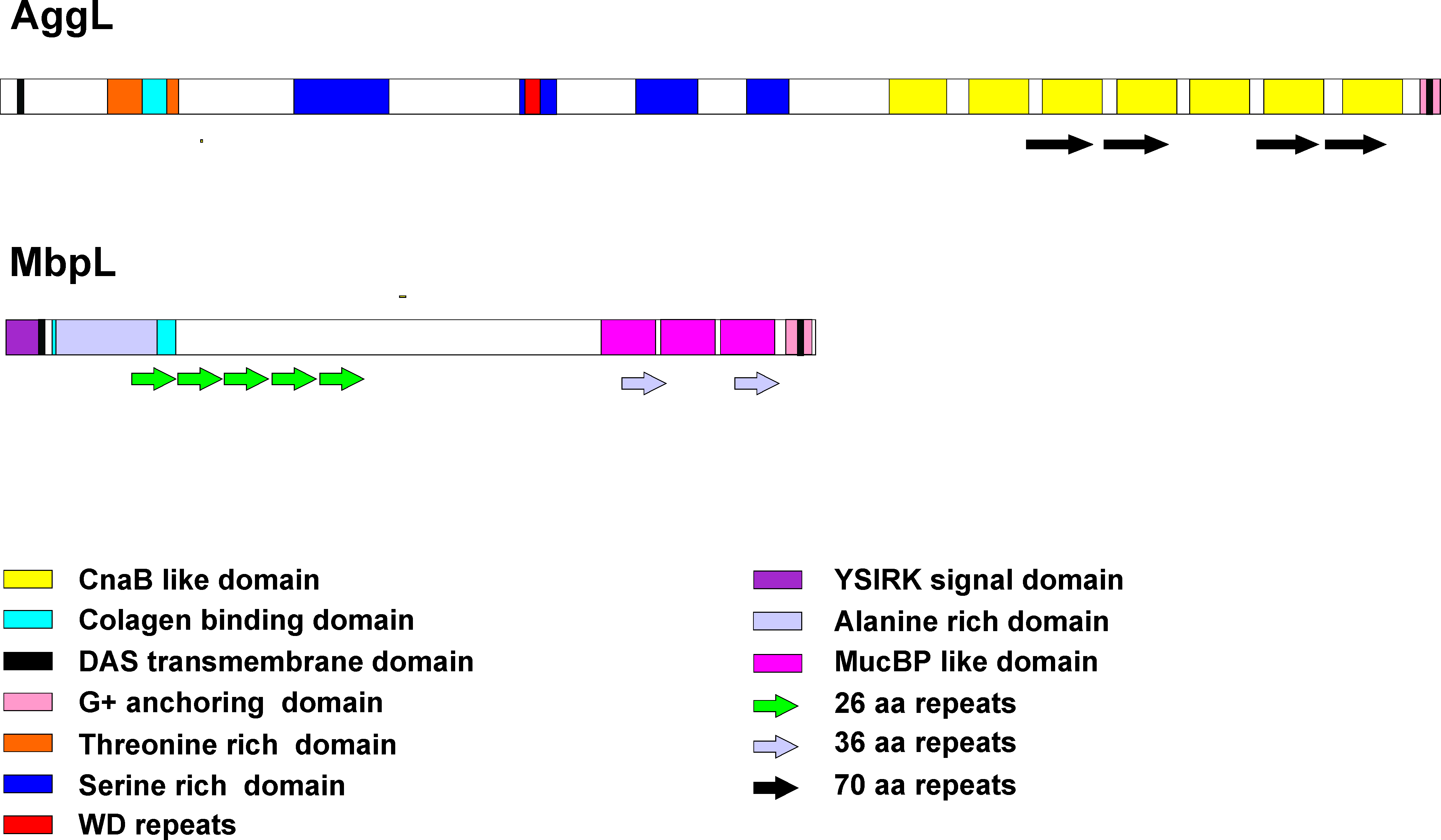

Supplement: Additional file 2 — Schematic presentation of AggL and MbpL proteins. Boxes indicate domains of proteins and arrows indicate repeats. [file 1471-2180-11-265-S2.TIFF]
